# A new species of *Nephus* (*Nephus*) (Coleoptera, Coccinellidae) described from Reunion Island

**DOI:** 10.3897/zookeys.962.51520

**Published:** 2020-08-20

**Authors:** Alexandra Magro, Julissa Churata-Salcedo, Emilie Lecompte, Jean-Louis Hemptinne, Lúcia M. Almeida

**Affiliations:** 1 Laboratoire Evolution et Diversité Biologique, UMR 5174 CNRS, UPS, IRD, 118 rt de Narbonne Bt 4R1, 31062 Toulouse cedex 9, France Laboratoire Evolution et Diversité Biologique Toulouse France; 2 ENSFEA, 2 rt de Narbonne, 31326 Castanet-Tolosan, France ENSFEA Castanet-Tolosan France; 3 Laboratório de Sistemática e Bioecologia de Coleoptera, Department of Zoology, Universidade Federal do Paraná, Caixa Postal 19030, 81581–980, Curitiba, Paraná, Brazil Universidade Federal do Paraná Curitiba Brazil; 4 Université Toulouse III-Paul Sabatier, 118 rt de Narbonne, Toulouse, France Université Toulouse III-Paul Sabatier Toulouse France

**Keywords:** Coccinelloidea, ladybird beetle, molecular data, predator, *
Scymnus
*, systematics

## Abstract

We report here a new species belonging to Nephus (Nephus) Mulsant. Nephus (Nephus) apolonia**sp. nov.** was collected in the Reunion Island (Mascarene Archipelago, Indian Ocean). We describe this new species and redescribe and illustrate three other *Nephus* species already known from Reunion: Nephus (Nephus) oblongosignatus Mulsant, 1850, Nephus (Geminosipho) reunioni (Fürsch, 1974) and Nephus (Nephus) voeltzkowi Weise, 1910. Furthermore, we present a phylogenetic tree for these four species and calculate the genetic distances between them, using high-throughput DNA sequencing of the mitochondrial genome. The similar external morphology of *N. apolonia***sp. nov.** and *N. voeltzkowi* very probably explains why individuals from the first species have been mistakenly identified as the latter and were not recognized as different until now. Other than external and genitalia traits, the present study provides molecular evidence confirming these are indeed two different species.

## Introduction

The Coccinellidae is a diversified family composed of some 6000 species, and the largest of the superfamily Coccinelloidea (Robertson et al. 2015). Seago et al. (2011) formally recognised two subfamilies within the Coccinellidae, Microweisinae and Coccinellinae*sensu*Ślipiński (2007). This last subfamily includes most of the Coccinellidae tribes, among them the Coccidulini*sensu*Seago et al. (2011), to which *Nephus* belongs. *Nephus* was first considered by Mulsant (1846) as a subgenus of *Scymnus* but Mader (1924) and then eventually Pope (1957) treated it as a valid genus.

The following characters distinguish *Nephus*: antennae with nine or pseudo-11 antenomeres; prosternal process sub-quadrangular, as wide as long, with a shallow lateral depression, without carina; legs with tarsi trimerous; abdomen with six ventrites, with incomplete postcoxal line, recurved and not reaching posterior margin of first ventrite.

According to Gordon (1976, 1985), *Nephus* has five subgenera: *Depressoscymnus* Gordon, *Nephus* Mulsant, *Scymnobius* Casey, *Sidis* Mulsant, and *Turboscymnus* Gordon; some of them have been considered as valid genera (Gordon and González 2002, Giorgi and González 2014). Fürsch (1987) described the subgenus Geminosipho and indicated the following species, Nephus (Geminosipho) bielawskii Fürsch, N. (Geminosipho) fenestratus (Sahlberg), N. (Geminosipho) koltzei (Weise). The same author (1987, 2007) considered eight *Nephus* subgenera: *Bipunctatus* Fürsch, 1987, *Depressoscymnus* Gordon, 1976, *Geminosipho* Fürsch, 1987, *Nephus* Mulsant, 1846, *Parascymnus* Chapin, 1965, *Scymnobius* Casey, 1899, *Sidis* Mulsant, 1850 and *Turboscymnus* Gordon, 1976.

Fürsch (2007), in his Catalogue of the African species of *Nephus*, reports 80 species belonging to four *Nephus* subgenera: *Nephus*, *Sidis*, *Geminosipho*, and *Bipunctatus*. Concerning specifically Reunion Island, Chazeau et al. (1974) mentioned three species, at that time considering *Nephus* as a subgenus of *Scymnus*: Scymnus (Nephus) voeltzkowi Weise, 1910, Scymnus (Nephus) oblongosignatus Mulsant, 1850 and Scymnus (Nephus) reunioni Fürsch, 1974. Poussereau et al. (2018) also mention these three species.

In this contribution, we describe a fourth Nephus (Nephus) species for Reunion Island, and redescribe the three already known species based on the study of a number of specimens of each species and using molecular data.

## Material and methods

The specimens examined here were provided by the first author from a laboratory rearing (Laboratoire Evolution & Diversité Biologique, Université Toulouse III) initiated from field collected material: *Nephus oblongosignatus* and *N. voeltzkowi* were collected in Reunion Island in 2011, *N. apolonia* sp. nov. was collected in Reunion Island in 2013, and *N. reunioni* was collected in 2007 in Portugal, where the species had been introduced for biological control in the 1980’s (Magro et al. 1999). The first author also observed specimens collected from 2006 to 2012 by the Insectarium of Reunion to investigate possible misidentifications of *N. apolonia* sp. nov. with *N. voeltzkowi* and to gather additional information on the geographical distribution of the new species on Reunion Island.

Photographs of the external morphology as well as male and female genitalia were taken using a Leica DMC 2900 Digital Camera attached to Leica M205C stereomicroscope using Leica Application Suite. Furthermore, specimens were examined with a JEOL JSM-6360LV scanning electron microscope in the Electronic Microscopy Center of Universidade Federal do Paraná. The length and width measurements of the species represent the average of the examined specimens.

The terminology used in the descriptions follows Ślipiński (2007). Labels of the type specimens are arranged in sequence from top to bottom, where the data for each label are within double quotes (“”), a slash (/) separates the rows, and information between brackets ([]) provides additional details written on the labels.

Examined material is deposited in the following collections: Coleção Entomológica Pe. J.S. Moure, Universidade Federal do Paraná, Curitiba, Paraná, Brazil (**DZUP**) and in Muséum National d’Histoire Naturelle, Paris, France (**MNHN**).

We used the mitochondrial genome of *Nephus* species previously sequenced by Magro et al. (2020): *N. reunioni*, *N. includens*, *N. voeltzkowi* and *N. apolonia* sp. nov. (voucher number: NeSpa1), together with the mitogenome of *N. oblongosignatus* sequenced in the present study (accession numbers: see Table 1) following the same protocol (see details in Magro et al. 2020).

Molecular characterization and distance analyses were conducted on the cytochrome c oxidase I (COI) gene using MEGA v.7 (Kumar et al. 2016). Pairwise distances were estimated between specimens using the Kimura-2-parameters model (Kimura 1980). We reconstructed the phylogenetic relationships between the *Nephus* species based on the mitogenome sequences (all protein coding and tRNA genes, but we deleted the control region because of the high divergence between species and the presence of repeated sequences, leading to low quality alignments in this region), using as outgroup the available sequence of *Cryptolaemus montrouzieri* which belongs to the same tribe as *Nephus* (i.e., Coccidulini*sensu*Seago et al. 2011) together with other Coccinellidae species (accession numbers: see Table 1). Sequences were aligned using MAFFT default parameters (Katoh and Standley 2013). We inferred maximum likelihood trees and bootstrapping with RAxML 8.2.10 (Stamatakis 2014) under the best-fitting model of sequence evolution for the dataset (GTR+G model), selected using the Akaike information criterion (AIC) using jModelTest 2 (Darriba et al. 2012).

**Table 1.** Genbank accession numbers for the mitogenome sequences used in the analysis.

Species	Genbank accession
*Nephus apolonia* sp. nov.	MN164644
*Nephus reunioni*	MN164643
*Nephus includens*	MN164642
*Nephus voeltzkowi*	MN164645
*Nephus oblongosignatus*	MT445723
*Propylea japonica*	KM244660
*Harmonia axyridis*	KR108208
*Cryptolaemus montrouzieri*	KT874575
*Henosepilachna pusillanima*	KJ131489

## Results and discussion

**Figure 1.**
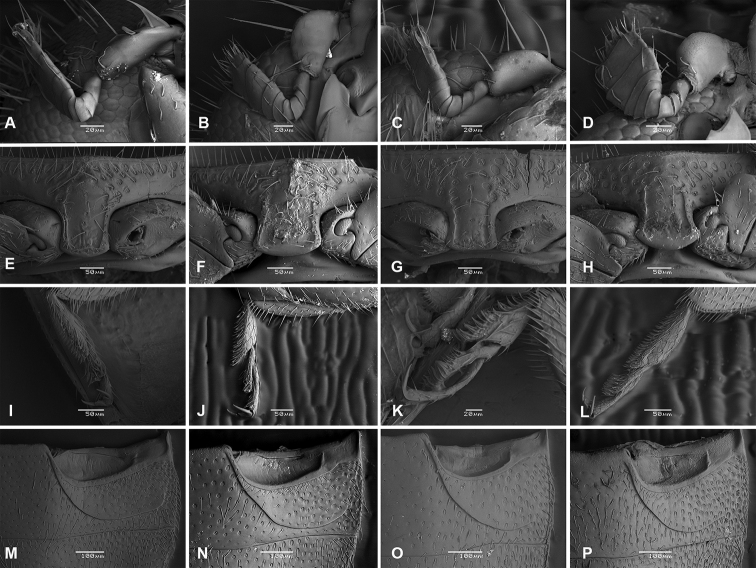
Scanning electron microscopy **A–D** antennae **E–H** prosternal process **I–L** tarsi **M–P** abdominal postcoxal line **A, E, I, M**Nephus (Nephus) oblongosignatus Mulsant, 1850 **B, F, J, N**Nephus (Geminosipho) reunioni (Fürsch, 1974) **C, G, K, O**Nephus (Nephus) voeltzkowi Weise, 1910 **D, H, L, P***Nephus apolonia* Magro & Almeida, sp. nov.

The species of *Nephus* present the following characteristics: antennae with nine or pseudo-11 antenomeres (Fig. 1A–D); prosternal process sub-quadrangular, as wide as long, with a shallow lateral depression, without carina (Fig. 1E–H); legs with tarsi trimerous (Fig. I–L); abdomen with six ventrites in males and females, with incomplete postcoxal line, recurved and not reaching posterior margin of the first ventrite (Fig. 1M–P).

### Key to species of *Nephus* from Reunion Island

**Table:** 

1	Each elytron black with one spot	**2**
1’	Each elytron black with two spots (Fig. 3)	**Nephus (Geminosipho) reunioni (Fürsch, 1974)**
2	Body rounded, oblong; each elytron with one yellowish oblong spot (Fig. 2)	**Nephus (Nephus) oblongosignatus Mulsant, 1850**
2’	Body elongated, each elytron with one yellowish elongated spot	**3**
3	Each elytron black with one big oval yellowish elongated spot, reaching middle of elytron; spermatheca with sharp base and truncated apex (Fig. 4)	**Nephus (Nephus) voeltzkowi Weise, 1910**
3’	Each elytron black with one small irregular yellowish spot, not reaching middle of elytron; spermatheca with sharp base and truncated apex (Fig. 5)	**Nephus (Nephus) apolonia Magro & Almeida, sp. nov.**

### Descriptions of species

#### Nephus (Nephus) oblongosignatus

Taxon classificationAnimaliaColeopteraCoccinellidae

Mulsant, 1850

42ED2DD7-CD06-5867-8BDD-648A7AF45570

Figs 1 , 2

Scymnus oblongosignatus Mulsant, 1850: 960 (original description).
Nephus oblongosignatus : Sicard 1909: 145–146; Weise 1910: 513.
Nephus grinerae Sicard, 1909: 145 (original description); Korschefsky 1931: 152 (synonymy).
Scymnus (Nephus) oblongosignatus : Korschefsky 1931: 152; Chazeau et al. 1974: 273 (systematics).
Nephus (Nephus) oblongosignatus : Poussereau et al. 2018: 130 (systematics).

##### Diagnosis.

*Nephus oblongosignatus* is similar to *N. voeltzkowi* and *N. apolonia* sp. nov. but differs in the body shape, size and shape of the spots and the pattern of genitalia.

##### Description.

**Figure 2.**
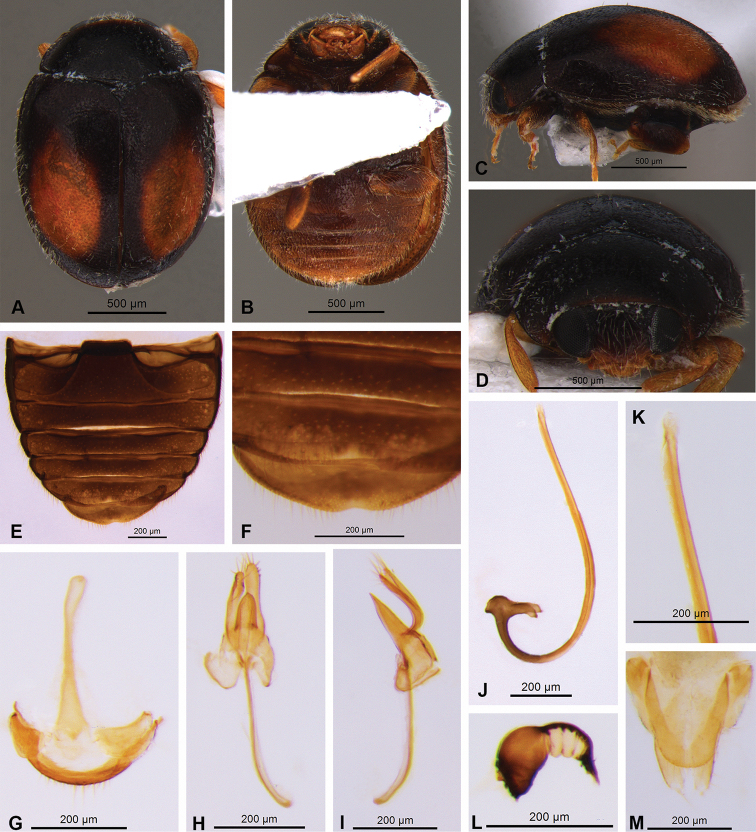
Nephus (Nephus) oblongosignatus Mulsant, 1850 **A** dorsal view **B** ventral view **C** lateral view **D** frontal view **E, F** abdomen **G–K** male genitalia: **G** spicula **H, I** tegmen (dorsal and lateral view) **J, K** penis **L, M** female genitalia: **L** spermatheca **M** coxites.

**Male.** Length 1.77 mm, width 1.28 mm. Body oval, oblong, with short fine whitish pubescence. Integument of pronotum, scutellar shield and elytra black (Fig. 2A). Elytra with one yellowish oblong spot on each elytron. Pronotum black, antero-lateral border dark brown (Fig. 2A, D). Head dark brown, antennae and mouthparts yellowish (Fig. 2B, D). Meso- and metaventrite light brown. Epipleuron light brown, without excavations to receive femora. Legs with coxae and femora dark brown, tibiae and tarsi light brown (Fig. 2B, C). Abdomen light brown; postcoxal line incomplete (Figs 1M, 2E) and last ventrite emarginated (Fig. 2F).

Genitalia with tegmen, penis guide, phallobase and parameres symmetrical. Spicule long (Fig. 2G). Penis guide shorter than parameres, sharp at apex (Fig. 2H, J). Parameres articulated with phallobase, distant from each other, strongly widened at apex, with long bristles along parameres (Fig. 2H, I). Penis sclerotized, J-shaped, with sharp apex, penis capsule T-shaped and elongated (Fig. 2J, K).

**Female.** Length 1.79 mm, width 1.34 mm. Similar to male. Genitalia with coxites longer than wide, subtriangular, 3.0× longer than wide; stylus mamiliform with long bristles (Fig. 2M). Spermatheca with thick walls, slightly arched, not very striated, and marked by one strong constriction in the middle; with sharp base and truncated apex (Fig. 2L).

##### Material examined.

Reunion Island: First generation from a laboratory rearing (Laboratoire Evolution & Diversité Biologique, Université Toulouse III) initiated from field material collected in November 2011 in Manapany-les-Bains, 19 specimens [DZUP].

#### Nephus (Geminosipho) reunioni

Taxon classificationAnimaliaColeopteraCoccinellidae

(Fürsch, 1974)

3F1296D6-EA0B-53C7-B29B-D44BF63FAEEF

Figs 1 , 3

Scymnus (Nephus) reunioni Fürsch, 1974: 275 (original description).
Nephus (Sidis) reunioni Fürsch 2007: 5 (systematics).
Nephus (Geminosipho) reunioni : Poussereau et al. 2018: 132 (systematics).

##### Diagnosis.

*Nephus reunioni* differs from the other species in the number, shape and size of the spots and the pattern of genitalia.

##### Description.

**Figure 3.**
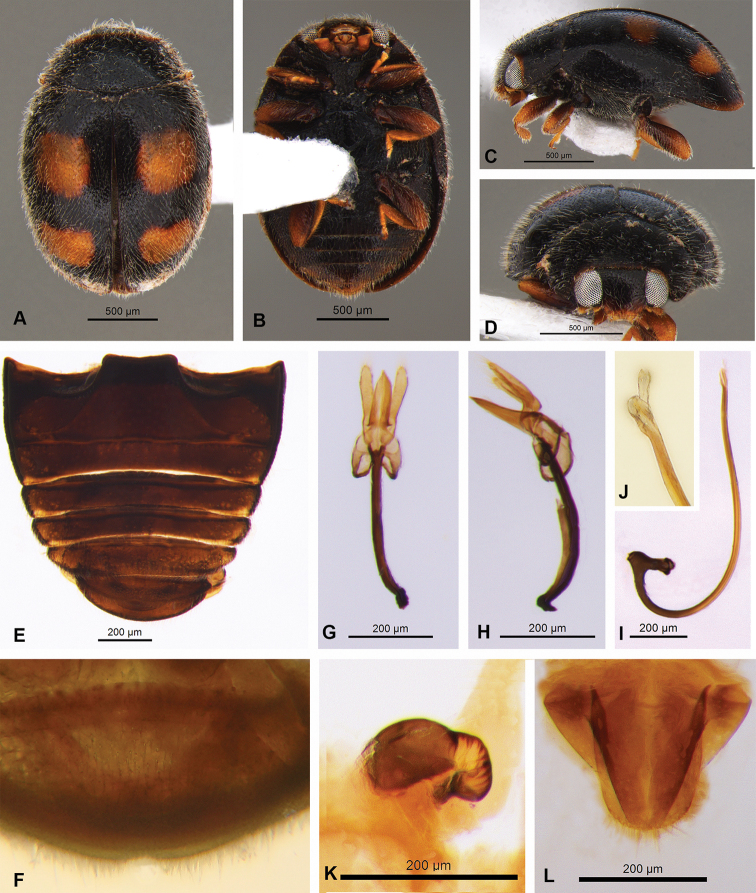
Nephus (Geminosipho) reunioni (Fürsch, 1974) **A** dorsal view **B** ventral view **C** lateral view **D** frontal view **E, F** abdomen **G–J** male genitalia: tegmen (dorsal and lateral view): **I, J** penis **K, L** female genitalia: **K** spermatheca **L** coxites.

**Male.** Length 1.7 mm, width 1.28 mm. Body oval, with short fine whitish pubescence. Integument of pronotum, scutellar shield and elytra black (Fig. 3A). Elytra with two yellowish transverse spots on each elytron, arranged in a row; elytra apex yellowish (Fig. 3A). Pronotum black, anterior border dark brown (Fig. 3A, D). Head dark brown, antennae and mouthparts yellowish (Fig. 3B). Meso- and metaventrite dark brown. Epipleuron black, without excavations to receive femora. Legs with coxae dark brown and femora, tibiae and tarsi yellowish (Fig. 1J). Abdomen dark brown; postcoxal line incomplete (Fig. 1N) and last ventrite emarginated (Fig. 3F).

Genitalia with tegmen, penis guide, phallobase and parameres symmetrical. Penis guide narrow, longer than parameres, sharp at apex. Parameres articulated with phallobase, distant from each other, strongly widened at apex, with long bristles along parameres (Fig. 3G, H). Penis sclerotized, J-shaped, with projection at apex, penis capsule T-shaped and elongated (Fig. 3J, I).

**Female.** Length 1.75 mm, width 1.30 mm. Similar to male. Genitalia with coxites longer than wide, subtriangular, 3.0× longer than wide; stylus mamiliform with short bristles (Fig. 3L). Spermatheca short, C- shaped, without ramus and nodulus, with sharp base and truncated apex (Fig. 3K).

##### Material examined.

Portugal: Specimens from a laboratory rearing (Laboratoire Evolution & Diversité Biologique, Université Toulouse III) initiated from field material collected in 2007 in Cascais, 10 specimens [DZUP].

##### Remarks.

It should be noted that Fürsch (2007), in his remarks about *N. reunioni*, mentions “The species is referred from various authors from South Africa (det. Fürsch), and even from Israel and Portugal. These specimens are breeded for pest control, but they seem to be misidentifications and in fact *N. derroni*.” In what concerns the Portugal population, we do not agree with Fürsch’s statement. Indeed, the specimens collected in Portugal and analyzed in the present study correspond to the original *N. reunioni* description by Fürsch presented in Chazeau et al. (1974). Raimundo (1992), who first described *N. reunioni* for Portugal, also illustrated the external morphology and genitalia corresponding to the original description by Fürsch in Chazeau et al. (1974). In both cases, the observations show that the specimens from the Portuguese population are distinct from *N. derroni*, first described from S. Tomé and presented in Fürsch (1974).

#### Nephus (Nephus) voeltzkowi

Taxon classificationAnimaliaColeopteraCoccinellidae

Weise, 1910

5600EB38-D185-5505-A8E9-CD728DF6BB04

Figs 1 , 4

Nephus (Nephus) voeltzkowi Weise, 1910: 512 (original description); Fürsch 2007: 6 (systematics).
Nephus seychellensis Sicard, 1912: 362 (original description); Chazeau et al. 1974: 272 (synonymy).
Scymnus (Nephus) voeltzkowi : Korschefsky 1931: 153 (catalog); Fürsch 1966: 181 (systematics).
Nephus (Nephus) voeltzkowi : Poussereau et al. 2018: 128 (systematics)

##### Diagnosis.

*Nephus voeltzkowi* resembles *N. oblongosignatus* and *N. apolonia* in the color of the integument and spots but differs in the shape and size of the spots and the female genitalia.

##### Description.

**Figure 4.**
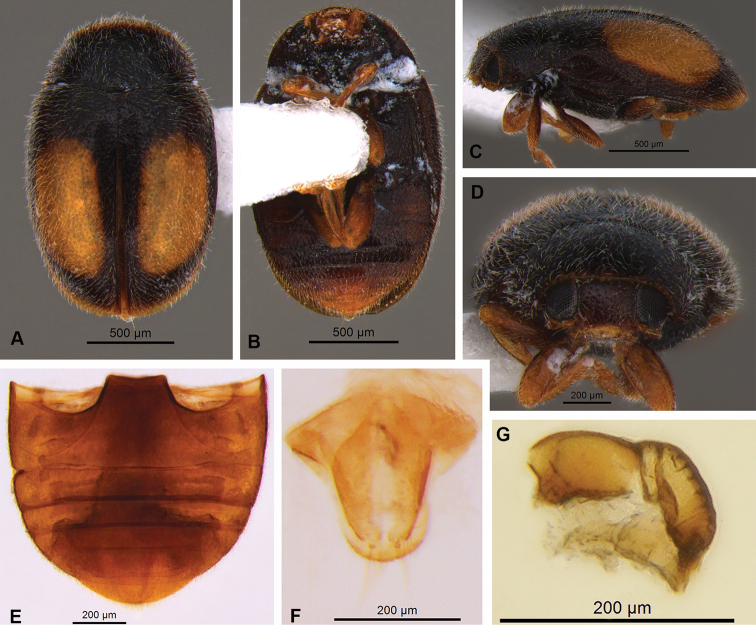
Nephus (Nephus) voeltzkowi Weise, 1910 **A** dorsal view **B** ventral view **C** lateral view **D** frontal view **E** abdomen **F, G** female genitalia: **F** coxites **G** spermatheca.

**Female.** Length 1.65 mm, width 1.10 mm. Body oval, with short fine whitish pubescence. Integument of pronotum, scutellar shield and elytra black. Elytra with one yellowish big oval spot on each elytron; elytra apex yellowish (Fig. 4A, C). Pronotum black, anterior border dark brown (Fig. 4A, D). Head, antennae and mouthparts light brown (Fig. 4B). Meso- and metaventrite dark brown. Epipleuron dark brown, without excavations to receive femora. Legs with coxae dark brown and femora, tibiae and tarsi light brown (Fig. 4B, D). Abdomen dark brown with two last ventrites yellowish; postcoxal line incomplete (Fig. 1O).

Genitalia. Coxites longer than wide, subtriangular, 3.0 x longer than wide; stylus mamiliform with long bristles (Fig. 4F). Spermatheca heavily sclerotized, slightly arched, striated, and marked by two strong constrictions in the middle; with sharp base and truncated apex (Fig. 4G).

Male genitalia according to Chazeau et al. (1974, plate II, figs 6, 7, 9, 10): tegmen, penis guide, phallobase and parameres symmetrical. Penis guide slightly longer than parameres. Parameres slender, articulated with phallobase, distant from each other, with bristles at apex. Penis sclerotized, J-shaped, with membranous apex, penis capsule T-shaped.

##### Material examined.

Reunion Island: Specimens from a laboratory rearing (Laboratoire Evolution & Diversité Biologique, Université Toulouse III) initiated from field material collected in November 2011 in Manapany-les-Bains, 14 specimens [DZUP].

##### Remarks.

It should be noted that only female specimens of *N. voeltzkowi* were observed here. Furthermore, although Magro et al. (2020) performed a large sampling campaign on Reunion, they never found *N. voeltzkowi* males, and eventually demonstrated that Reunion females are parthenogenetic. As indicated by Magro et al. (2020), the presence of *N. voeltzkowi* was reported before by Chazeau et al. (1974) in their fauna of ladybirds from Reunion, but the sex of the specimens was not mentioned: although Chazeau et al. (1974) presented an illustration of the genitalia of a *N. voeltzkowi* male, the possibility that the drawing was based on a Madagascar specimen was not discounted (Chazeau pers. com.). In the absence of the original material, we cannot confirm this information.

#### Nephus (Nephus) apolonia

Taxon classificationAnimaliaColeopteraCoccinellidae

Magro & Almeida sp. nov.

9B108A65-D25A-5DA1-9B9B-2893541DB5D9

http://zoobank.org/9CCCB544-1EE3-4F28-AD13-4359F999AE33

Figs 1 , 5

##### Diagnosis.

*Nephus apolonia* sp. nov. is similar to *N. voeltzkowi* and *N. oblongosignatus* but differs by the size and shape of the spots and the pattern of genitalia.

##### Description.

**Figure 5.**
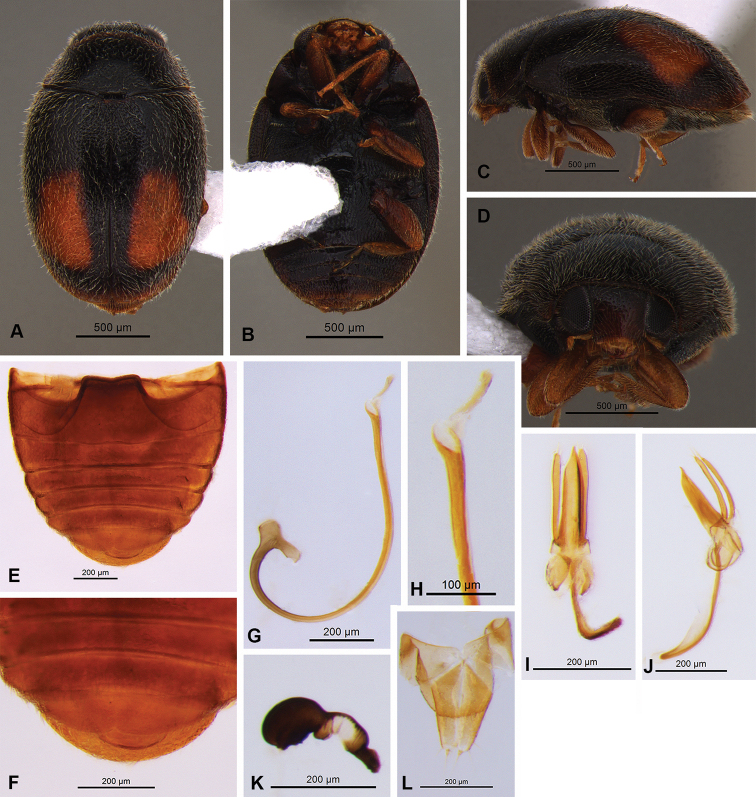
Nephus (Nephus) apolonia Magro & Almeida, sp. nov. **A** dorsal view **B** ventral view **C** lateral view **D** frontal view **E, F** abdomen **G–J** male genitalia: **G, H** penis **I, J** tegmen (dorsal and lateral view) **K, L** female genitalia: **K** spermatheca **L** coxites.

**Male.** Length 1.69 mm, width 1.2 mm. Body oval, oblong, with short fine whitish pubescence. Integument of pronotum, scutellar shield and elytra black. Elytra with one yellowish longitudinal spot on each elytron (Fig. 5A, C). Pronotum black, antero-lateral border dark brown (Fig. 5A, D). Head dark brown, antennae and mouthparts yellowish (Fig. 5B, D). Meso- and metaventrite light brown. Epipleuron light brown, without excavations to receive femora. Legs with coxae and femora dark brown, tibiae and tarsi light brown (Figs 1L, 5B, D). Abdomen light brown (Fig. 5E); postcoxal line incomplete (Fig. 1P), and last ventrite emarginate (Fig. 5F).

Genitalia with tegmen, penis guide, phallobase and parameres symmetrical. Penis guide shorter than parameres, sharp at apex (Fig. 5I, J). Parameres articulated with phallobase, distant from each other, strongly widened at apex, with short bristles along parameres (Fig. 5I, J). Penis sclerotized, J-shaped, with sharp apex, penis capsule T-shaped and elongated (Fig. 5G, H).

**Female.** Length 1.88 mm, width 1.30 mm. Similar to male. Genitalia with coxites longer than wide, subtriangular, 3.0× longer than wide; stylus mamiliform with long bristles (Fig. 5L). Spermatheca heavily sclerotized, slightly arched, not very striated, and marked by one strong constriction in the middle, with sharp base and rounded apex (Fig. 5K).

##### Etymology.

This species is named after an early name of Reunion Island, mentioned as “Santa Apolonia” on the Portolan charts (nautical charts) from the XVI^th^ century (GENUNG, 2017).

##### Type locality.

Reunion Island: from a laboratory rearing (Laboratoire Evolution & Diversité Biologique, Université Toulouse III) initiated from field material collected in December 2013 in Manapany-les-Bains and Étang-Salé.

##### Type material.

***Holotype*** male, pinned, with genitalia in a separate microvial. Original label: “Reunion Island, 1 specimen [MNHN]”; “HOLOTYPE/ *Nephus apolonia* Magro and Almeida” [red label]. ***Paratypes*.** The following specimens are designated as paratypes with labels: “same data as for holotype”. “PARATYPE/ *Nephus apolonia* Magro and Almeida” [yellow label]: “Reunion Island, 2 specimens [MNHN, DZUP]; “Reunion Island, 1 specimen [MNHN]; “Reunion Island, 1 specimen [DZUP]; “Reunion Island, 1 specimen [MNHN]; “Reunion Island, 1 specimen [DZUP]; “Reunion Island, 2 specimens [DZUP, MNHN]; “Reunion Island, 1 specimen [DZUP].

##### Geographical distribution.

Reunion Island: L’Étang-Salé, Le Trou d’eau (21°16'54.2"S, 55°21'39.7"E); Saint-Denis, Saint-Bernard (20°52'58.36"S, 55°23'50.19"E); Saint-Louis, Etang du Gol (21°17'20.9"S, 55°23'16.1"E); La Possession, Ravine à Malheur (20°54'03.5"S, 55°22'32.1"E); Saint-Pierre, CIRAD (21°19'13.8"S, 55°29'6"E); L’Étang-Salé, ARDA (21°17'05.6"S, 55°22'38.1"E) and Saint-Joseph, Langevin (21°22'53.4"S, 55°38'48.4"E).

##### Remarks.

*Nephus apolonia* sp. nov. has apparently been misidentified as *N. voeltzkowi* until now. We verified that this was the case for specimens captured by the Insectarium de La Réunion. Poussereau et al. (2018) included three species for Reunion Island. In that work, *N. voeltzkowi* is reported with large variation and distribution. It could be possible that the specimens identified by Poussereau et al. (2018) as *N. voeltzkowi* included the *Nephus apolonia* sp. nov. described here.

##### Molecular analysis.

**Figure 6.**
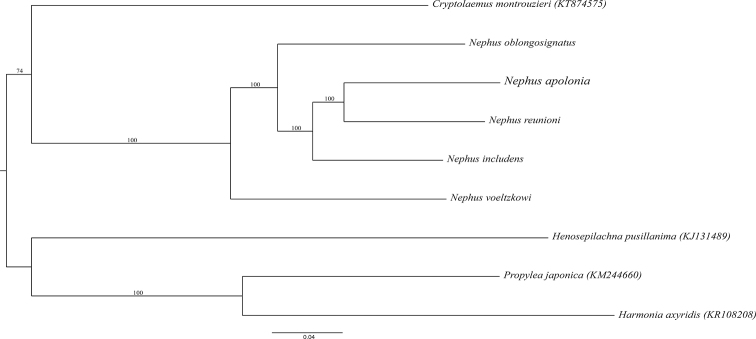
Maximum likelihood phylogeny of Nephus (Nephus) apolonia Magro & Almeida, sp. nov. and four related species based on the mitochondrial genome (without the control region) reconstructed through 1000 non-parametric bootstrap replicates. The scale bar indicates 0.04 substitutions per site. Numbers on major nodes represent Maximum Likelihood bootstrap support.

The mitochondrial genome of one specimen of *Nephus apolonia* is deposited in Genbank under accession number MN164644. Genetic distances based on the COI sequences between *N. apolonia* and other *Nephus* species confirm that *N. apolonia* is different from the other species, as all distances are within the same range (i.e., 0.13–0.17) (Table 2). In the phylogenetic tree reconstructed from 14,867 pb of aligned mitochondrial genomes (Fig. 6), most nodes, including *N. apolonia*, were supported by high bootstrap values.

**Table 2.** Pairwise Kimura-2-parameter distances for the mitochondrial COI gene for the *Nephus* species.

		**1**	**2**	**3**	**4**
**1**	*Nephus apolonia*				
**2**	*N. reunioni*	0.131			
**3**	*N. includens*	0.133	0.126		
**4**	*N. voeltzkowi*	0.174	0.170	0.160	
**5**	*N. oblongosignatus*	0.155	0.150	0.142	0.172

## Supplementary Material

XML Treatment for Nephus (Nephus) oblongosignatus

XML Treatment for Nephus (Geminosipho) reunioni

XML Treatment for Nephus (Nephus) voeltzkowi

XML Treatment for Nephus (Nephus) apolonia
